# Quality More Than Quantity: The Use of Carbohydrates in High-Fat Diets to Tackle Obesity in Growing Rats

**DOI:** 10.3389/fnut.2022.809865

**Published:** 2022-03-29

**Authors:** Manuel Manzano, Maria D. Giron, Rafael Salto, Jose D. Vilchez, Francisco J. Reche-Perez, Elena Cabrera, Azahara Linares-Pérez, Julio Plaza-Díaz, Francisco Javier Ruiz-Ojeda, Angel Gil, Ricardo Rueda, Jose M. López-Pedrosa

**Affiliations:** ^1^Abbott Nutrition R&D, Granada, Spain; ^2^Department of Biochemistry and Molecular Biology II, School of Pharmacy, University of Granada, Granada, Spain; ^3^Instituto de Investigación Biosanitaria de Granada (ibs.GRANADA), Complejo Hospitalario Universitario de Granada, Granada, Spain; ^4^Children’s Hospital of Eastern Ontario Research Institute, Ottawa, ON, Canada; ^5^Biomedical Research Center, Institute of Nutrition and Food Technology “José Mataix,” University of Granada, Granada, Spain; ^6^CIBEROBN Physiopathology of Obesity and Nutrition, Institute of Health Carlos III, Madrid, Spain

**Keywords:** obesity, slow digestive carbohydrates, metabolism, lipidomic analysis, growing rats

## Abstract

Childhood obesity prevention is important to avoid obesity and its comorbidities into adulthood. Although the energy density of food has been considered a main obesogenic factor, a focus on food quality rather that the quantity of the different macronutrients is needed. Therefore, this study investigates the effects of changing the quality of carbohydrates from rapidly to slowly digestible carbohydrates on metabolic abnormalities and its impact on obesity in growing rats fed a high-fat diet (HFD). Growing rats were fed on HFD containing carbohydrates with different digestion rates: a HFD containing rapid-digesting carbohydrates (OBE group) or slow-digesting carbohydrates (ISR group), for 4 weeks and the effect on the metabolism and signaling pathways were analyzed in different tissues. Animals from OBE group presented an overweight/obese phenotype with a higher body weight gain and greater accumulation of fat in adipose tissue and liver. This state was associated with an increase of HOMA index, serum diacylglycerols and triacylglycerides, insulin, leptin, and pro-inflammatory cytokines. In contrast, the change of carbohydrate profile in the diet to one based on slow digestible prevented the obesity-related adverse effects. In adipose tissue, GLUT4 was increased and UCPs and PPARγ were decreased in ISR group respect to OBE group. In liver, GLUT2, FAS, and SRBP1 were lower in ISR group than OBE group. In muscle, an increase of glycogen, GLUT4, AMPK, and Akt were observed in comparison to OBE group. In conclusion, this study demonstrates that the replacement of rapidly digestible carbohydrates for slowly digestible carbohydrates within a high-fat diet promoted a protective effect against the development of obesity and its associated comorbidities.

## Introduction

The global prevalence of childhood obesity is increasing in developed and developing countries; it is considered the biggest public health challenge of the 21st Century. The World Health Organization reports that over 340 million children and adolescents aged 5–19 presented overweight or obesity in 2016. The increase was similar in boys and girls: in 2016, 18% of girls and 19% of boys were overweight (1). The rapid transition from underweight to overweight and obesity children has been identified as a relevant public health problem with social consequences (2), especially in developing countries.

Overweight and obesity in children are linked to serious short- and long-term complications. Studies have shown that overweight and obesity children are likely to retain their weight into adult life, increasing the incidence of non-communicable diseases and the risk of morbidity and premature death in adulthood (1, 3). Furthermore, children with obesity could have future risks: breathing problems, increased risk of fractures, hypertension, and early markers of cardiovascular disease, insulin resistance, and psychological effects (4).

Obesity is a complex multifactorial disease that affects all the organs in the body. It is characterized by irregular or excessive fat accumulation, as a consequence of an imbalance between energy intake and energy expenditure combined with a genetic predisposition for weight gain (2). Epidemiological studies have identified a correlation between dietary fat intake and obesity and its related complications (5). In addition, studies in children aged 5–7 years, and followed up to 15 years, found that the dietary pattern consisting of a diet high in energy density, fats, and sugars and scarce in fiber, fruits, and vegetables was associated with higher percentage of body fat and an excess of adiposity in childhood and adolescence (6, 7). Other factors in the lifestyle of children, such as insufficient physical activity and excessive sedentarism, and food consumption of high-calorie sweetened beverages, have also been related to the increase of childhood obesity (8, 9).

The high energy density of food has been considered an obesogenic factor. However, several studies using energy-dense foods have failed to establish a relationship with weight gain, leading to the suggestion that the obesogenic capacity of foods may be due to their composition rather than their energy density. In addition to reducing calorie intake, an alternative would be to identify the role of certain nutrients and how they should be distributed in diets. For this, diets have been proposed for the prevention or treatment of obesity that uses different sources of carbohydrates (CHO), proteins, and fats combined in turn in different proportions. However, preventing obesity may need to focus on the quality rather than the quantity of the different macronutrients.

Therefore, behavioral strategies to decrease caloric intake, decrease sedentary lifestyle, and increase physical activity are the key for pediatric weight management. The standard approach in treating obesity involves reducing dietary fat, the most energy-dense nutrient. However, the weight loss characteristics of a reduced-fat diet are moderate and transient (10). Recently, dietary CHO have been proposed as a target for the treatment of obesity.

Carbohydrates are essential for growth and development and one of the main sources of energy in infancy and childhood. As indicated previously (8), an increase in the consumption of added sugars is associated with increased obesity in children. Concerning the quality of CHO, epidemiological studies have suggested different measures for their evaluation, the main criteria being the consumption of whole grains, the intake of dietary fiber, the solid or liquid form of CHO, and the glycemic index (GI) (11). GI classifies CHO-containing foods based on the postprandial glucose response and represents the quality of CHO. A higher GI produces a faster rise in the postprandial serum glucose and rapid insulin response. The fast response to insulin leads to rapid hypoglycemia, combined with hunger and therefore, a higher caloric intake. On the contrary, a low GI diet translates into a slower absorption of CHO and less blood glucose fluctuations reflecting a better glycemic control (12, 13). An adult study shows how the consumption of slow-digesting sugars such as isomaltulose in comparison to sucrose can help with weight management by reducing fat. In contrast, studies of pre-adolescent children have revealed that when high-glycemic CHO are consumed for breakfast, the amount of food consumed during lunch is greater. In summary, these previous studies indicate that the intake of different types of CHO [i.e., rapid-digested versus slow-digested CHO (ISR)] not only affects the blood sugar response but also affects subsequent energy intake (14). In terms of animals, previous studies have shown that the mother’s consumption of ISR induce a greater metabolic flexibility later on in the growth and development of the offspring, regulating lipid metabolism, improving muscle functionality, and reducing predisposition to liver disease (15–17). However, studies conducted in humans have reported conflicting results in the relationship between GI and obesity, suggesting the importance of not only quantity but also the CHO profile of the diet (18).

In the present study, we investigate the effects of changing the quality of CHO from rapidly digestible CHO to slowly digestible CHO on metabolic abnormalities and its impact on overweight and obesity in growing rats upon high-fat diet (HFD) feeding. Our working hypothesis is that the quality of CHO that have a low GI and sustained glucose release (such as isomaltulose and Sucromalt®) and prebiotic effects (such as resistant maltodextrin, inulin, and fructooligosaccharides) might prevent these metabolic alterations. In our study, the main results clearly showed that the replacement of rapid digestible CHO for slowly digestible CHO (ISR) in a HFD prevented the HFD-obesity-related adverse effects and improve lipid metabolism and glucose control through different mechanisms including regulation of hormones, such as insulin and leptin, and incretins (GLP-1), as well as the management of cellular metabolic pathways related to lipogenesis and protein homeostasis.

## Materials and Methods

### Housing

Thirty weanling male Wistar Han International Genetic Standard (IGS) rats (21–25 days old) were provided by Charles Rivers (Orleans Cedex, France). Animals were individually housed in cages and kept under 12 h light-12 h dark cycles. The room temperature was maintained at 21°C.

All experimental procedures (approval code 29/10/2018/152) were carried out according to the European Convention for the Protection of Vertebrate Animals used for Experimental and other Scientific Purposes (Council of Europe No 123, Strasbourg 1985) as well as to the ethical guidelines for animal experimentation provided by the Spanish National Research Council (RD 1201/2005 October 10).

### Experimental Design

An HFD-induced animal model has been considered as an appropriate model to study dietary obesity. This animal model is adequate to study the mechanisms by which dietary fat influences the regulation of energy balance (14). Post-weaning rats in a HFD also presented a phenotype of metabolic syndrome and increased hepatic steatosis (11).

Rats (*n* = 10/group) were randomly assigned to three nutritional groups based on the diet received:

(i) NOB, a lean group. Rats were fed on a standard rodent diet (AIN93G) (19).

(ii) OBE, an obese group. Rats were fed on an HFD (20).

(iii) OBE-ISR, an obese group. Rats were fed on an HFD formulated with ISR.

All diets were formulated to have the same amount of total fiber. In addition, both HFD were designed to be isoenergetic. All information regarding the diets is listed in Table 1.

**TABLE 1:** Composition of diets.

Macronutrients	AIN93G	HF	HF-ISR
CHO (g/100 g diet)	64.59	49.42	49.42
Sucrose (g/100 g CHO)	14.37	36.07	
Isomaltulose (g/100 g CHO)			26.40
Sucromalt (g/100 g CHO)			22.10
Cornstarch (g/100 g CHO)	50.66	23.50	
Maltodextrins (g/100 g CHO)	18.97	24.43	34.50
Resistant starch (g/100 g CHO)			10.00
Inulin:FOS (g/100 g CHO)			7.00
Cellulose (g/100 g CHO)	16.00	16.00	
*Total sugar* (g/100 g CHO)	*15.00*	*36.85*	*39.10*
*Total fiber* (g/100 g CHO)	*16.00*	*16.00*	*16.00*
*Glycemic load*	*726*	*687*	*338*
Protein (g/100 g diet)	17.00	24.19	24.19
Fat (g/100 g diet)	7.00	20.00	20.00
Energy (calories/100 g diet)	372.13	458.63	458.63

*HF, high-fat diet; RDC, diet with rapidly digestible CHO; ISR, diet with slowly digestible CHO; CHO, carbohydrates; Inulin:FOS, 1:1 mixture of fructooligosaccharides (FOS).*

*Glycemic load estimates the glycemic index of the carbohydrate blend resulting from the sum of each component glycemic index multiplied by its amount in the diet.*

The nutritional intervention was conducted for 4 weeks. All rats had free access to food and water, and animal weight and food consumption were determined weekly. Body composition, body fat mass, and lean body mass were measured at baseline and the end of the nutritional treatment by quantitative nuclear magnetic resonance imaging (EchoMRI 700 system; Echo Medical Systems, Houston, TX, United States). After the feeding period, rats were introduced to a Phenomaster Indirect Calorimetry System (TSE System, Bad Homburg, Germany) for analyzing energy expenditure.

At the end of the study, animals were euthanized in post-absorptive conditions [1 h after an oral meal tolerance challenge with their corresponding experimental diets; 10 kcal diet/kg of body weight (BW)]. Blood samples were taken before (12 h fasting) and after oral gavage. Blood was collected either into serum tubes or in tubes containing anticoagulant EDTA (for isolating plasma). Tissues were immediately isolated, weighed, and snap-frozen in liquid nitrogen and kept at −80°C for posterior analysis.

### Biochemical Parameters

A hemogram was analyzed using the hematology analyzer Pentra 80 (Horiba ABX, Montpellier, France). Serum/plasma was collected and parameters such as glucose, triacylglycerols (TG), cholesterol, low-density lipoprotein (LDL)-cholesterol, high-density lipoprotein (HDL)-cholesterol, and non-esterified fatty acids (NEFA) were analyzed using a clinical chemistry analyzer Pentra 400 (Horiba ABX, Montpellier, France). Insulin and leptin concentration in serum, as well as serum interleukin-(IL)-6, IL-1β, monocyte attractant proteins 1 (MCP-1), and tumor necrosis factor α (TNF-α) were measured by Bio-Plex 200 system (Bio-Rad, Hercules, CA, United States). The assays were performed on a 96-well plate according to product instructions. Glucagon-like peptide-1 (GLP-1) secreted was measured using an ELISA kit (Mercodia, Uppsala, Sweden) according to the manufacturer’s instructions. The Homeostatic Model Assessment index of insulin resistance (HOMA-IR) was calculated as fasting glucose (mmol/L) × fasting insulin (mU/L)/22.5 (21).

### Lipidomics Analysis

The concentration of serum TG was measured using a TG-LQ kit (Spinreact, Barcelona, Spain). Metabolite profiles were analyzed as previously described (22, 23). Concisely, the semi-quantification of lipid species was performed using two separate UHPLC-time-of-flight (TOF)–MS-based platforms (Agilent Technologies, Santa Clara, CA, United States) and, afterward, analyzing the combination of methanol and chloroform-methanol liver extracts. Non-esterified fatty acids, bile acids, and lysoglycerophospholipids were evaluated in the methanol extract platform. The chloroform-methanol extract platform allowed the analysis of glycerolipids, glycerophospholipids, sterol lipids, and sphingolipids. This combined analysis was established for rodent serum by OWL Metabolomics (Derio, Spain). Data obtained with the UHPLC–MS were processed with the TargetLynx application manager for MassLynx 4.1 (Waters Corp., Milford, MA, United States). Intra- and inter-batch normalization followed the procedure published by Martinez-Herranz et al. (24). All the calculations were performed with R v3.2.0 (R Development Core Team, Vienna, Austria, 2010).

### Western Blot Analysis

Tissue samples were lysed using a 50 mM HEPES pH 7.5, 150 mM NaCl, 1% Nonidet P-40, 10 mM NaF, 20 mM NaPPi, 1 mM MgCl_2_, 1 mM CaCl_2_, 20 mM β-glycerophosphate, 2 mM sodium orthovanadate, 2 mM EDTA, 2 mM PMSF, 4 μg/l leupeptin buffer. Tissues were homogenized for 15 s in Polytron at setting #4. The homogenate was centrifuged at 16,000 × *g* for 15 min at 4°C. The supernatant was transferred to a new Eppendorf tube (1.5 mL) and sonicated for 15 s (cycle 0.5, amplitude 70%). Rat gastrocnemius muscle was pulverized using a mortar and liquid nitrogen before being subjected to the above homogenization protocol. The protein concentration of the samples was measured using the bicinchoninic acid method (25). Specific antibodies against GLUT4 (Biogenesis Ltd., Poole, United Kingdom); total and phospho (Ser473)-PKB/Akt, total and Phospho-AMPKα 1/2 (Thr172), and MEF2D (Cell Signaling, Beverly, MA, United States); creatine kinase, carbohydrate-responsive element-binding protein (ChREBP), fatty acid synthase (FAS), glucose transporter 2 (GLUT2), sterol regulatory element-binding protein-1 (SREBP1), Pyruvate Kinase (PKM1/2), Pyruvate dehydrogenase kinase (PDK4), ATPase5B, uncoupling protein (UCP)-1, and UCP-2 (Santa Cruz Biotechnology, Dallas, TX, United States) were used. Glyceraldehyde 3-phosphate dehydrogenase (GAPDH) (Santa Cruz Biotechnology) was used as load control. Data were normalized using the values of the reference animals as 100%.

### Glycogen Quantification

Hepatic and muscle glycogen was isolated as described (26). Tissue homogenates (10%) were prepared in 0.03 N HCl and spread evenly on pieces of filter paper (Whatman 3M chromatography paper, 2.0 × 2.0 cm) in duplicate. The papers were gently stirred in a beaker containing 66% EtOH. Then, papers were washed three times for 40 min in 66% EtOH, rinsed with acetone, and dried. The dried filter papers were cut into four pieces and placed in a tube containing 0.4 mL of 0.2 M acetate buffer, pH 4.8; 0.2 mg of amylo-α-1,4-α-1,6-glucosidase; and H_2_O to a final volume of 2 mL. samples were incubated for 90 min at 37°C with gentle shaking. Controls were prepared by incubating aliquots of homogenate in acetate buffer minus amyloglucosidase. Glucose content in the incubated samples was determined by the glucose oxidase method.

### Statistical Analysis

Results were expressed as means ± SEM. The statistical significance of variations was evaluated using one- or two-way ANOVA or the corresponding non-parametric test depending on the homoscedasticity test (Bartlett’s test). *Post hoc* paired comparisons, using Tukey’s test or Dunn’s test, were performed to check for significantly different effects between all pairs of diets using the GraphPad Prism 8.0 software. A *p*-value < 0.05 was considered significant.

## Results and Discussion

The present preclinical study aimed to evaluate the effects of a specialized CHO diet that differs in quality rather than in the quantity of CHO (rapid digestible vs. slowly digestible CHO) to prevent excessive fat deposition in a well-established HFD rodent model of childhood obesity induced by feeding a HFD. Since metabolic syndrome in children and adolescents has increased dramatically in recent years, animal studies using younger rats may be of interest, to apply the experimental results in the pediatric population. Thus, the use of rats after weaning could allow mimicking the childhood condition. Rats fed an HFD after weaning developed the main characteristics of the metabolic syndrome: central obesity, systolic and diastolic hypertension, altered fasting glucose, hypertriglyceridemia, and decreased HDL cholesterol levels (27). The nutritional intervention was carried out for 4 weeks from weaning to infancy. A non-obese control group was termed NOB in which the animals were fed *ad libitum* with a standard growth diet AIN93G for rodents. The model used has proven to be valid to decide the time of nutritional intervention in rodents to adapt their metabolism and to avoid obese syndrome.

**FIGURE 1:**
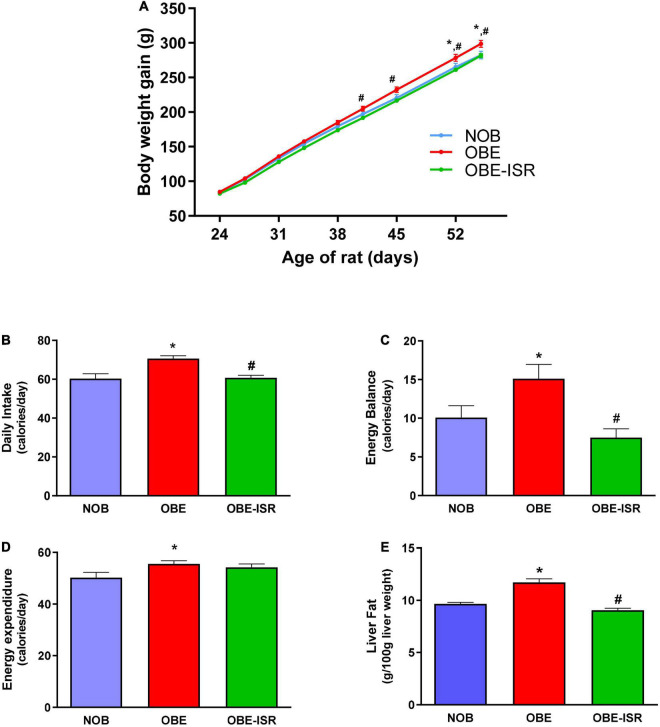
Growth and energy expenditure in the experimental model. **(A)** Body Weight evolution. **(B)** Daily intake. **(C)** Energy balance. **(D)** Energy Expenditure. **(E)** Liver fat. Rats were fed a normal rodent diet (NOB), or a high-fat diet (HFD) formulated with rapidly (OBE) or with slowly digestible carbohydrates (OBE-ISR). Data expressed as mean ± SEM. *Significant difference with NOB group, *p* < 0.05. #Significant difference with OBE group, *p* < 0.05.

After 4 weeks, consumption of HFD promoted a significantly higher weight gain than rats in the lean group from the third week of nutritional intervention (Figure 1). In contrast, rats fed on the OBE-ISR diet showed a similar pattern to the lean group, with significantly less weight gain compared to the OBE group (Figure 1A). As previously mentioned, obesity is the consequence of a maintained positive energy balance over time. It has been reported in humans that a very small deviation from energy balance, on the order of 1–2% of daily energy, can increase body weight by approximately 20 kg (28). In our study, rats fed on the OBE diet had a higher daily intake than the other groups (Figure 1B), while energy expenditure was also greater than the lean group (Figure 1C). Therefore, the energy balance of the OBE rats was approximately 15 calories/day, more than 5 calories/day compared to lean. In contrast, the OBE-ISR group showed an energy balance similar to the lean group (Figure 1D).

A positive energy balance drives excess lipid accumulation in adipose tissue (29). The OBE group showed significant differences regarding the percentage of total body fat compared to the NOB and OBE-ISR groups (Table 2). The OBE group presented an increase of up to 20% in fat mass. The increase in fat tissue was mainly due to a higher accumulation in visceral and subcutaneous depots. However, there were no differences in the lean body mass among dietary groups, and hind-leg muscle weights were also similar (Table 2). Hence, the results obtained from the OBE group indicated that the increase in body weight gain during the experimental period rendered an increase in fat tissue without gain of lean body mass and, in contrast, this effect was not observed in the OBE-ISR group.

**TABLE 2:** Body composition and serum biochemistry of the different experimental groups fed on the different experimental diets for 4 weeks to induce obesity.

	NOB	OBE	OBE-ISR
Final fat body mass (g)	25.85 ± 2.12	31.79 ± 2.27*	25.75 ± 1.02^#^
Final lean body mass (g)	233.6 ± 5.1	245.5 ± 4.8	233.5 ± 3.4
Visceral WAT depots (g)	10.18 ± 2.78	14.31 ± 0.61*	10.72 ± 0.84^#^
Subcutaneous WAT depots (g)	6.32 ± 032	8.12 ± 0.56*	5.99 ± 0.30^#^
Hind-leg muscle (g)	2.19 ± 0.08	2.38 ± 0.08	2.38 ± 0.05
Fasting serum TG (mg/dL)	62.59 ± 10.90	81.39 ± 6.12	48.49 ± 5.92^#^
Fasting serum cholesterol (mg/dL)	68.15 ± 5.05	75.56 ± 4.72	75.39 ± 6.55
Fasting serum LDL-cholesterol (mg/dL)	6.23 ± 0.64	6.93 ± 0.48	7.73 ± 0.82
Fasting serum HDL-cholesterol (mg/dL)	24.56 ± 1.32	25.96 ± 1.23	27.53 ± 1.93
Fasting serum NEFA (mmol/L)	0.83 ± 0.08	0.71 ± 0.08	0.59 ± 0.05
TG/HDL ratio	2.20 ± 0.40	2.96 ± 0.37	1.44 ± 0.14^#^
Fasting serum glucose (mg/dL)	109.5 ± 9.3	130.0 ± 8.8	130.0 ± 8.1
Fasting serum insulin (pg/mL)	615.3 ± 143.7	788.0 ± 166.2	556.3 ± 90.8
HOMA-IR index	3.63 ± 1.11	7.28 ± 1.41*	4.56 ± 0.79^#^
Fasting serum leptin (mg/dL)	2175 ± 429	3667 ± 474*	1597 ± 275^#^
Postprandial serum GLP-1 (pmol/L)	2.294 ± 0.127	2.778 ± 0.280	4.514 ± 0.267*^#^

*Data expressed as mean ± SEM (n = 10).*

*Significant difference with NOB group, p < 0.05.

*#Significant difference with OBE group, p < 0.05.*

*WAT, white adipose tissue; TG, triacylglycerols; LDL, low-density lipoprotein; HDL, high-density lipoprotein; NEFA, non-esterified fatty acids; GLP-1, glucagon-like peptide-1.*

Obesity-related complications are associated with alterations in TG storage from adipocytes and the release of fatty acids (FA), leading to dysfunctional adipose tissue and triggering lipodystrophy (30). These individuals show a poor ability to recruit adipocytes to healthy subcutaneous locations in response to increased energy intake. Instead, they store excess fat in ectopic deposits, such as liver or visceral fat, which are directly associated with the metabolic dysregulation of obesity. In the liver, this metabolic imbalance can lead to a condition known as non-alcoholic fatty liver disease (22), which represents the most common cause of chronic liver disease in children and adolescents (31). Our results showed that 4 weeks of feeding with the OBE diet significantly increased the presence of fat in the liver compared to the NOB group. In contrast, the group that was fed an OBE-ISR diet did not increase liver fat accumulation, maintaining normal values (Figure 1E).

In addition, other obesity-related biomarkers such as serum TG, cholesterol, LDL, HDL, and NEFA were analyzed in fasting (Table 2). HFD produced an increase in serum TG compared to the other groups, while no differences in cholesterol, LDL-c, HDL-c or NEFA were found in the dietary groups. High levels of circulating TG are a feature in obese children and adolescents, and at the same time, hypertriglyceridemia is a known biomarker of cardiometabolic risk. Observational studies suggest that cardiovascular disease is caused by high levels of circulating TG and TG-rich lipoproteins. Recently, the TG/HDL-cholesterol ratio has been proposed as a marker of structural vascular changes in obese young people (30) better than the TG or cholesterol levels. In our experimental setting, rats from the OBE-ISR showed the lowest ratio compared to the other two groups (Table 2), indicating that changing the CHO profile could reduce the risk of developing cardiovascular disease in adulthood.

**FIGURE 2:**
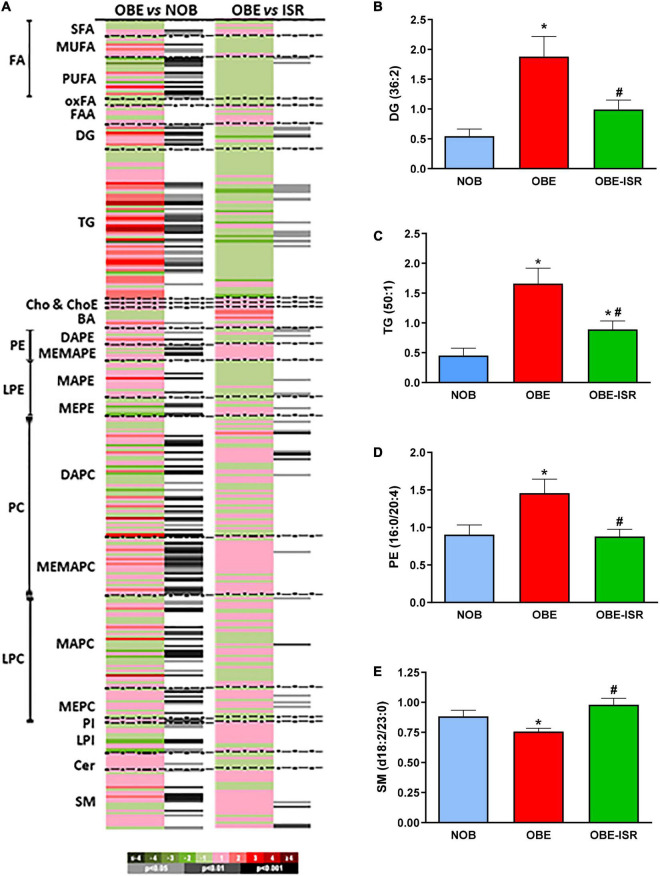
Lipidomic analysis of the plasma in the experimental groups. **(A)** Heat map showing relevant changes in plasma. Each metabolite is shown as a line whose color is defined by the sign and magnitude of the change. Adjacent column to each comparison shows the results of the *t*-test. Relevant lipid species of **(B)** Diacylglycerols, **(C)** Triglycerides, **(D)** Phosphatidylethanolamines, **(E)** Sphingomyelins of the different experimental groups fed on the different experimental diets for 4 weeks to induce obesity. Data expressed as mean ± SEM. *Significant difference with NOB group, *p* < 0.05. #Significant difference with OBE group, *p* < 0.05. Cer, ceramides; ChoE, cholesterol esters; DAG, diacylglycerols; FFAox, oxidized free fatty acids; LPC, lysophosphatidylcholines; LPE, lysophosphatidylethanolamines; PC, phosphatidylcholines; PE, phosphatidylethanolamines; PI, phosphatidylinositols; MUFA, monounsaturated fatty acids; PUFA, polyunsaturated fatty acids; SFA, saturated fatty acids; SM, sphingomyelins; TG, triacylglycerols.

To further explore the metabolic changes induced by the diets during the growing period, a global lipidomics approach was applied in serum. A total of 361 metabolic features were individually semi quantified in fasting serum. Both OBE and OBE-ISR experimental groups led to several changes in the serum metabolic profile when compared to the lean group. Results were plotted as a heatmap in Figure 2A. The OBE group showed high levels of monounsaturated fatty acids, diacylglycerols (DG), TG, and phosphoethanolamine (PE), while the sphingomyelin (SM) group was decreased (Figures 2B–E). These results observed in the OBE group would be associated with an obesogenic profile as they would be in line with the changes described in obese individuals (32). In obesity, levels of lysophosphatidylcholines, ceramides, SM and total fatty acids are increased, whereas ethanolamine and lysoPE are decreased (32–35). In contrast to OBE group, when the OBE-ISR group was analyzed, the lipid profile was not so affected, showing a metabolic profile close to the lean group (Figures 2B–E).

Both body fat mass and lipid profile have been considered to significantly increase the risk of later development of diabetes (32). Children with obesity exhibit increased lipid deposition in the visceral and intra-myocellular compartments, which causes severe insulin resistance (36). In our study, fasting glucose and insulin levels were measured (Table 2). Although there were no changes in blood glucose between the groups, the animals fed the OBE diet tended to show hyperinsulinemia compared to the OBE-ISR group (*p* = 0.10). Next, the HOMA index was calculated as a measure of insulin resistance and the highest value was observed in the HFD group, with significant differences to NOB and OBE-ISR groups (Table 2). These results are consistent with different randomized controlled trials in children and adolescents, which described the positive effect of a low GI/glycemic load (GL) diet on insulin resistance compared to a high GI/GL dietary approach (37).

In addition to insulin, peripheral hormones, such as leptin and GLP-1, play central roles in the central control of energy metabolism. Leptin is considered an antiobesity hormone that controls body weight and fat accumulation through its interaction with hypothalamic receptors, and its level is related to body fat mass. Among its main actions are appetite inhibition, metabolic rate stimulation and thermogenesis. In fasting conditions, a decrease in leptin levels is observed while they increase after feeding, following a mechanism that helps to regulate energy balance in humans. However, several studies indicate the existence of an endogenous mechanism of leptin resistance in obesity (38, 39). Our results in the OBE group agree with the hyperleptinemia described in obese individuals, reaching statistical differences to the NOB group. In contrast, the HFD supplemented with ISR in the OBE-ISR group led to leptin levels similar to those obtained in the lean control group (Table 2), indicating an improvement of the putative leptin resistance in the rats.

Glucagon-like peptide-1 is an incretin synthesized and released by intestinal L-cells. Nutrient intake is the primary stimulus for GLP-1 secretion, which increases its levels two-threefold after a meal. It has been shown that GLP-1 suppresses appetite in both normal and obese individuals, playing a major role in energy metabolism regulation. Additionally, GLP-1 improves glucose control, increases insulin sensitivity and stimulates insulin secretion. Therefore, the role of GLP-1 in the regulation of the metabolic disorders induced by obesity is crucial. However, some studies suggest that GLP-1 secretion and signaling are reduced in obese adolescents (40). In our experimental design, postprandial GLP-1 secretion was not affected by the obesogenic diet, showing similar levels in NOB and OBE groups (Table 2). Nonetheless, postprandial GLP-1 levels in the OBE-ISR group were significantly higher than in the other two groups. This effect could be related to the CHO profile of the diet since it has been described that GLP-1 secretion is affected by the GI. Thus, CHOs that are more slowly digested are likely to be exposed to more distal intestinal regions, with correspondingly later, but potentially more substantial, GLP-1 secretion (40).

Overall, the OBE-ISR diet generated an increase in GLP-1 and a decrease in leptin with respect to the HF diet, which indicates its positive effect on both appetite control and satiety and other metabolic processes such as glucose control.

In addition to the above, adipose tissue expansion in obesity could drive adipocyte dysfunction, low-grade systemic inflammation, and ultimately insulin resistance. Indeed, adipose tissue is not only a site of energy storage but also an active metabolic/endocrine organ involved in the secretion of adipokines, chemokines and pro-inflammatory cytokines. The association between childhood overweight and low-grade systemic inflammation was based on a large cross-sectional analysis (NHANES III) (41). In this study, serum inflammatory markers and white blood cell counts were significantly higher in overweight than in lean children, indicative of an inflammation state. In this way, a significant increase in leukocytes, especially in monocytes, was observed when rats were fed the OBE diet in comparison with the NOB group (Table 3). Along with this, an increase in the levels of pro-inflammatory cytokines in postprandial serum was observed, such as IL-6, IL-1β, TNF-α, and MCP-1 (Table 3). These results would be in agreement with those described in the process of inflammation in obesity, where an infiltration of macrophages into adipose tissue can be due to the migration of monocytes from the bloodstream by the action of a chemoattractant agent. The increase of macrophages in adipose tissue would increase the secretion of inflammatory cytokines, which is negative feedback would increase the state of inflammation (42, 43). When data for OBE-ISR groups were analyzed, there were no differences in comparison to the lean control group (Table 3). These results would indicate the prevention of HFD-associate adipocyte metabolic dysfunction by the CHO profile in this diet.

**TABLE 3:** Serum immunological markers of the different experimental groups fed on the different experimental diets for 4 weeks to induce obesity.

	NOB	OBE	OBE-ISR
Blood leukocytes (10^3^/mm^3^)	9.88 ± 0.775	13.10 ± 1.46*	11.18 ± 0.50^#^
Blood lymphocytes (%)	72.58 ± 0.89	73.31 ± 3.07	73.92 ± 2.08
Blood monocytes (10^3^/mm^3^)	0.224 ± 0.026	0.331 ± 0.030*	0.221 ± 0.025^#^
Serum IL-6 (pg/mL)	243.6 ± 70.9	2203.0 ± 1121.0*	550.2 ± 51.0*^#^
Serum IL-1β (pg/mL)	12.64 ± 2.37	20.30 ± 3.33*	15.20 ± 1.24^#^
Serum TNF-α (pg/mL)	4.69 ± 0.96	5.60 ± 0.76	5.30 ± 0.92
Serum MCP-1 (pg/mL)	703.2 ± 157.1	980.6 ± 185.8	767.8 ± 75.63

*Data expressed as mean ± SEM (n = 10).*

*Significant difference with NOB group, p < 0.05.

*#Significant difference with OBE group, p < 0.05.*

*IL, interleukin; TNF, tumor necrosis factor; MCP-1, monocyte chemoattractant protein 1.*

The results obtained up to this point show that the animals fed a HFD and rapidly digestible CHO presented an overweight/obese phenotype with a higher body weight gain and greater accumulation of fat in adipose tissue and liver. This state was associated with a lipid imbalance and insulin resistance, as well as a low-grade inflammatory state. In contrast, the change of CHO profile in the diet to one based on ISR prevented metabolic imbalance and the animals, even with a HFD and the same quantity of CHO, presented a phenotype close to the lean group fed a standard growth diet. Considering these results, we analyze the different cellular pathways involved in these metabolic processes in adipose tissue, liver and muscle to determine the mechanisms of action underlying this effect.

**FIGURE 3:**
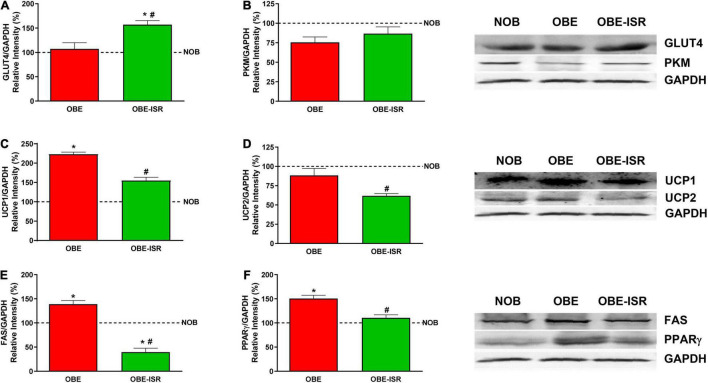
Adipose tissue expression of GLUT4, PKM, uncoupling proteins and lipid synthesis related proteins in the experimental groups. **(A)** GLUT4. **(B)** PKM. **(C)** UCP1. **(D)** UCP2. **(E)** FAS. **(F)** PPARγ. Rats were fed a normal rodent diet (NOB) or a high-fat diet (HFD) formulated with rapidly (OBE) or with slowly digestible carbohydrates (OBE-ISR). Data expressed as mean ± SEM. *Significant difference with NOB group, *p* < 0.05. #Significant difference with OBE group, *p* < 0.05.

Metabolically, adipose tissue is characterized by the storage of TG and the withdrawal of glucose from the blood in the postprandial period in a process dependent on the insulin-dependent GLUT4 transporter. Insulin resistance states, such as diabetes and obesity, are associated with lower expression of GLUT4 in adipose tissue (44–46). Here, GLUT4 protein levels were significantly higher in the OBE-ISR group than in the OBE and NOB groups (Figure 3A), which would indicate an improvement of insulin resistance in the OBE-ISR group compared to the OBE group, according with HOMA data (Table 2). In addition, insulin resistance provokes changes in the flow of adipocyte substrates, releasing of FA and glycerol (47). We observed higher levels of blood FA in the NOB (31.20 ± 2.24) and OBE (30.92 ± 2.94) compared with OBE-ISR (23.9 ± 1.02) group. In terms of improvement of insulin sensitivity due to the observed effects on GLUT4 in adipose tissue and HOMA, the glycolytic use of the glucose was measured as the expression of PKM1/2 and we did not find differences between groups (Figure 3B). On the other hand, the uncoupling proteins (UCP1 and UCP2) catalyze proton leakage across the mitochondrial membrane, causing uncoupling of the electron transport chain and ATP synthesis (48). UCP1, which was first discovered in brown adipose tissue, has a relevant role in thermogenesis and body weight control. Furthermore, an increase in the expression of UCP1 due to low temperatures or overfeeding has been described. UCP2 is a mitochondrial transporter that plays a key role in the regulation of glucose/lipid metabolism and energy homeostasis. UCP2 downregulation increases insulin resistance in white adipose tissue (49). The OBE-ISR rats showed no changes in the expression of the ATP synthase (data not shown), although the levels of UCP1 and UCP2 were significantly lower in the OBE-ISR group compared to the OBE rats (Figures 3C,D), which could be interpreted in terms of better functionality of these mitochondria for the aerobic oxidation of substrates.

The OBE group presented an increase in visceral adiposity compared to the OBE-ISR, and it was evidenced by the analysis of enzymes and transcription factors related to lipogenesis. The expression of fatty acid synthase (FAS), a key enzyme implicated in *the novo* synthesis of FA in adipose tissue, was significantly higher in the OBE than in the other groups (Figure 3E). Furthermore, in adipose tissue, adipogenesis is regulated by transcription factors such as peroxisome proliferator-activated receptors (PPARs). In adipose tissue, insulin sensitivity and adipogenesis are controlled by PPARs, transcription factors whose expression is also regulated by alterations in glucose and lipid homeostasis (50). Among the different PPARs, PPARγ regulates the expression of genes involved in the endogenous synthesis of cholesterol, fatty acids, and triacylglycerols (51). Our results indicate that the OBE diet stimulated the expression of PPARγ and would be an additional factor in the increased adiposity observed in these rats. In contrast, the ISR diet decreased PPARγ expression to levels comparable to the lean control group (Figure 3F).

Overall, in adipose tissue, the OBE-ISR diet increased GLUT4 levels, which would be associated with better insulin sensitivity. However, the higher insulin sensitivity was not associated with greater adiposity since our data indicate that FA synthesis and the expression of lipogenesis-related transcription factors (PPARγ) are decreased in these rats.

The liver provides energy and substrates to other tissues, being responsible for the homeostasis of glucose and other metabolic fuels (52). Thus, it participates in the processing of dietary fats, proteins and CHO. It also plays an important role in glucose metabolism either to obtain energy in the form of ATP or metabolic intermediates that will be substrates for the synthesis of glycogen or TG (53, 54).

The diet OBE is associated with an increase in the levels of serum TG (Table 2) and also with higher liver fat (Figure 1E). These circulating TG come from hepatic synthesis. Here, we analyzed the uptake of glucose and fatty acids into the liver through the expression of specific transporters, the flow of these substrates toward their storage as glycogen or TG, as well as the regulation of these processes were analyzed. To do this, the phosphorylative state of Akt, as the kinase responsible for insulin sensitivity in liver cells and transcription factors such as ChREBP and SRBP1, whose expression is associated with higher lipid synthesis, were measured.

**FIGURE 4:**
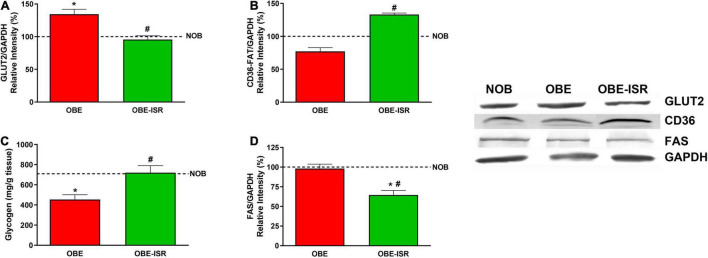
Uptake and storage of metabolic substrates in liver. **(A)** GLUT2. **(B)** CD36-FAT. **(C)** Glycogen. **(D)** FAS. Rats were fed a normal rodent diet (NOB) or a high-fat diet (HFD) formulated with rapidly (OBE) or with slowly digestible carbohydrates (OBE-ISR). Data expressed as mean ± SEM. *Significant difference with NOB group, *p* < 0.05. #Significant difference with OBE group, *p* < 0.05.

The incorporation of CHO with a high GI to a HFD led to higher protein levels of the glucose transporter, GLUT2, and a decrease of the fatty acid transporter expression, CD36, in the liver of animals that received this diet (Figures 4A,B). Concomitantly, the greater amount of glucose that has entered the liver of OBE animals does not translate into an increase in glycogen synthesis (Figure 4C), being this group the one with the lowest levels of glycogen. As suggested by Irimia et al. (55), we can speculate that inability to synthesize liver glycogen is caused by CHO with a high GI induced lipid synthesis and liver fat over-accumulation, accompanied by impairment in hepatic insulin sensitivity. Both the reference (AIN93G) and the HFD are rich in fast-digesting CHO. These CHO can be converted into fatty acids, as indicated by the expression of FAS, which is higher in these two groups than in the OBE-ISR one (Figure 4D). The changes in FAS expression in adipose tissue and liver were associated with circulating FA and TG levels in fasted animals and also with higher lipid storage in the own liver. In states of insulin resistance, the increased flow of FA to the liver is the result of a failure of insulin to suppress TG lipolysis in adipose tissue.

Fatty acids are packaged as TG into VLDL particles and transported to adipose and other extrahepatic tissues. The presence of hepatic fat in the OBE group could indicate that the hepatic synthesis of FA might be greater than its capacity to secrete VLDL; therefore, they remain stored. It has been described that in obese individuals or those with insulin resistance, there is an increase in lipid synthesis. Furthermore, many obese subjects have normal plasma VLDL-TG levels, whereas those with high blood VLDL-TG levels have an increased VLDL secretion rate. Although plasma VLDL-TG levels could be higher in obese than in lean individuals, hepatic TG accumulation is mainly caused by an imbalance between hepatic TG synthesis and TG export *via* VLDL (56–58).

In the liver, the glycogen content of the OBE-ISR group is higher than in the other two experimental groups, indicating a relevant role for the liver in glycemic control in response to this complex CHO-enriched diet (Figure 4C). Another positive effect of the ISR diet is that hepatic FAS showed a significant decrease compared to the OBE and lean groups (Figure 4D). These results indicated a shift in liver CHO metabolism toward glycogen synthesis in the OBE-ISR group rather than the use of glucose as a lipogenic substrate.

**FIGURE 5:**
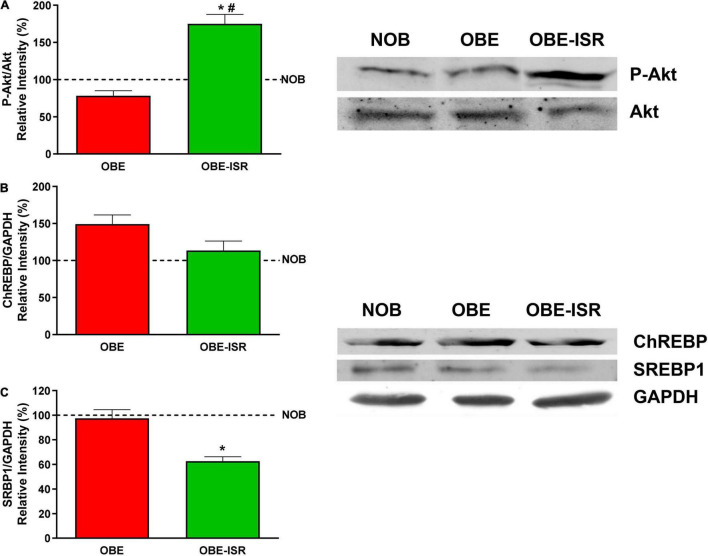
Key signaling elements related to insulin sensitivity and lipid synthesis in the liver. **(A)** p-Akt. **(B)** ChREBP. **(C)** SRBP1. Rats were fed a normal rodent diet (NOB) or a high-fat diet (HFD) formulated with rapidly (OBE) or with slowly digestible carbohydrates (OBE-ISR). Data expressed as mean ± SEM. *Significant difference with NOB group, *p* < 0.05. #Significant difference with OBE group, *p* < 0.05.

The key signaling elements measured in the liver confirmed this behavior. Animals that were fed the OBE-ISR showed a significant increase in phosphorylated Akt/PKB kinase (Figure 5A), indicating again that the composition of CHO can reverse the impact of a HFD on the sensibility to insulin. Furthermore, the regulation of lipogenesis in the liver is mainly dependent on the expression of ChREBP and SREBP1 proteins (59). SREBP1c regulates most of the genes involved in the synthesis of FA and TG: acetyl-CoA carboxylase, FAS, elongases, and acyl-CoA desaturases. ChREBP is mainly regulated by glucose but plays a complementary role with SREBP1 in the regulation of hepatic lipogenesis. Our results indicate a decrease in these transcriptional regulators levels in the OBE-ISR group when compared to the OBE one preventing lipogenic metabolic programming (Figures 5B,C).

**FIGURE 6:**
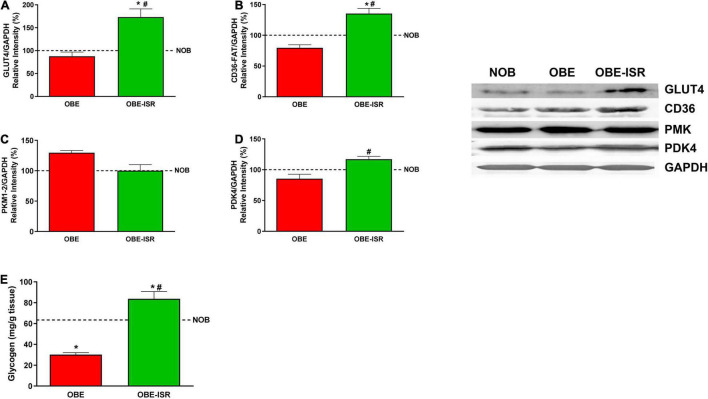
Uptake and metabolism of glucose and fatty acids in the muscle. **(A)** GLUT4. **(B)** CD36-FAT. **(C)** PKM1/2. **(D)** PDK4. **(E)** Glycogen. Rats were fed a normal rodent diet (NOB) or a high-fat diet (HFD) formulated with rapidly (OBE) or with slowly digestible carbohydrates (OBE-ISR). Data expressed as mean ± SEM. *Significant difference with NOB group, *p* < 0.05. #Significant difference with OBE group, *p* < 0.05.

Finally, skeletal muscle contributes to maintaining a functional metabolism. Obesity and type 2 diabetes mellitus impaired glycogen synthesis in the skeletal muscle could be a side effect of skeletal muscle insulin resistance of glucose transport. The insulin-dependent glucose transporter, GLUT4, was overexpressed in the OBE-ISR group compared to the NOB and OBE control groups (Figure 6A). The higher expression of GLUT4 transporter in the OBE-ISR group would be related to a better sensitivity to the action of insulin. The increase in glucose uptake in the OBE-ISR group was in parallel with an increase in the muscle glycogen content in this group while the glucose that entered the muscle of the OBE rats was utilized by the glycolytic pathway (Figure 6E). Furthermore, the FA transporter, CD-36, was also overexpressed in the OBE-ISR group compared to the NOB and OBE control groups (Figure 6B) indicating higher oxidation of the FA that constitutes the preferred fuel of the muscle at rest (60). Another interesting feature of muscle function in rats that received the OBE-ISR diet can be obtained from the measurement of proteins that regulate the oxidation of fuels or the differentiation of muscle cells, as well as the activation of related signaling pathways. The pyruvate dehydrogenase kinase isoenzyme 4 (PDK4) is a protein responsible for controlling fuel use in muscles. An increase in the PDK4 expression is associated with phosphorylation/inhibition of the pyruvate dehydrogenase complex. In this situation, the pyruvate is not converted to acetyl-CoA and is not used for fatty acid synthesis. The OBE-ISR group showed a higher PDK4 expression than the OBE one (Figure 6D). Therefore, the OBE-ISR could oxidize the FA that came through the CD36 transport to obtain energy. In addition, the inhibition of the pyruvate dehydrogenase complex by PDK4 observed in this group would lead to an inhibition of the entry of pyruvate into the Krebs cycle, it would block glucose oxidation (52), which would be in agreement with the lower expression of PKM in the OBE-ISR group (Figure 6C).

**FIGURE 7:**
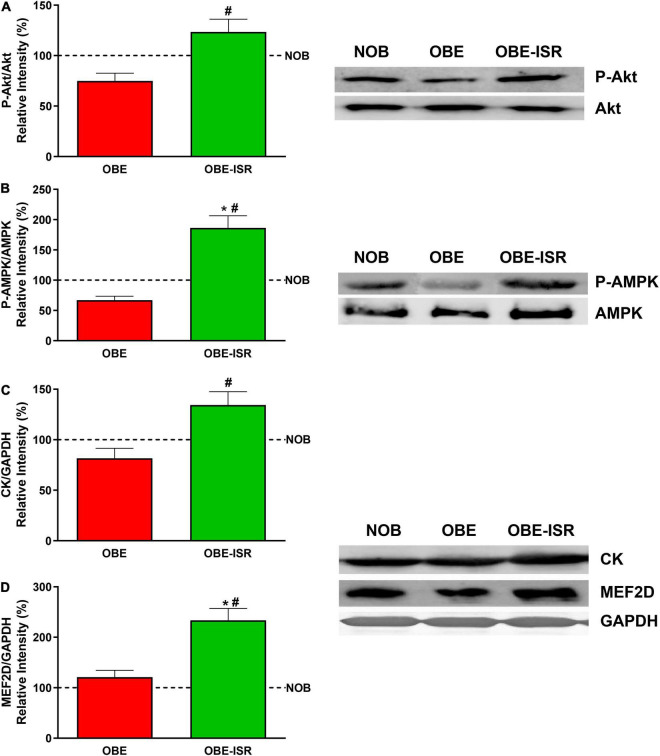
Activation of key signaling elements and expression of differentiation markers in the muscle. **(A)** p-Akt. **(B)** p-AMPK. **(C)** CK. **(D)** MEF2D. Rats were fed a normal rodent diet (NOB) or a high-fat diet (HFD) formulated with rapidly (OBE) or with slowly digestible carbohydrates (OBE-ISR). Data expressed as mean ± SEM. *Significant difference with NOB group, *p* < 0.05. #Significant difference with OBE group, *p* < 0.05.

Our results also revealed that the phosphorylation of Akt and AMPK is higher in the OBE-ISR rats than in the other groups (Figures 7A,B). The activation of Akt pointed out to an increase in the sensibility to insulin and that of AMPK is related to increased glucose uptake in muscle and FA oxidation in muscles at rest (61).

Finally, despite the fact that the lean body mass was not increased in the OBE-ISR group, the muscles of these animals had increased expression of creatine kinase and MEF2D (Figures 7C,D), which translated into better muscle functionality and differentiation compared to the other groups (62).

## Conclusion

In this work focused on evaluating the effect of CHO on the development of obesity in childhood, replacement of rapidly digestible CHO by ISR in an obesogenic HF diet promotes a significant protective effect against the development of obesity and its associated comorbidities. The quality of the diet exerted its effect on energy balance through complex hormonal and cellular pathways such as lipogenesis, cellular energy, insulin sensitivity and inflammation. Overall, the presence of these CHO in the diet in the childhood stage would preserve the lean phenotype in the individual and prevent the appearance of obesity and its associated comorbidities in adulthood.

## Data Availability Statement

The raw data supporting the conclusions of this article will be made available by the authors, without undue reservation.

## Ethics Statement

The animal study was reviewed and approved by the Consejería de Agricultura, Ganaderia, Pesca y Desarrollo Sostenible, Junta de Andalucia, Spain.

## Author Contributions

MM, MDG, RR, and JL-P: conceptualization and supervision. MM, RS, MDG, JV, FR-P, EC, and AL-P: data curation and investigation. RR and JL-P: funding acquisition. RS, MDG, JL-P, and MM: writing—original draft. RS, MDG, MM, JL-P, JP-D, FR-O, and AG: writing—review and editing. All authors have read and agreed to the published version of the manuscript.

## Conflict of Interest

This study received funding from Abbott Laboratories S.A. MM, RR, and JL-P as Abbott employees have had the following involvement with the study: Study design, data collection and analysis, decision to publish, and preparation of the manuscript. The remaining authors declare that the research was conducted in the absence of any commercial or financial relationships that could be construed as a potential conflict of interest.

## Publisher’s Note

All claims expressed in this article are solely those of the authors and do not necessarily represent those of their affiliated organizations, or those of the publisher, the editors and the reviewers. Any product that may be evaluated in this article, or claim that may be made by its manufacturer, is not guaranteed or endorsed by the publisher.
